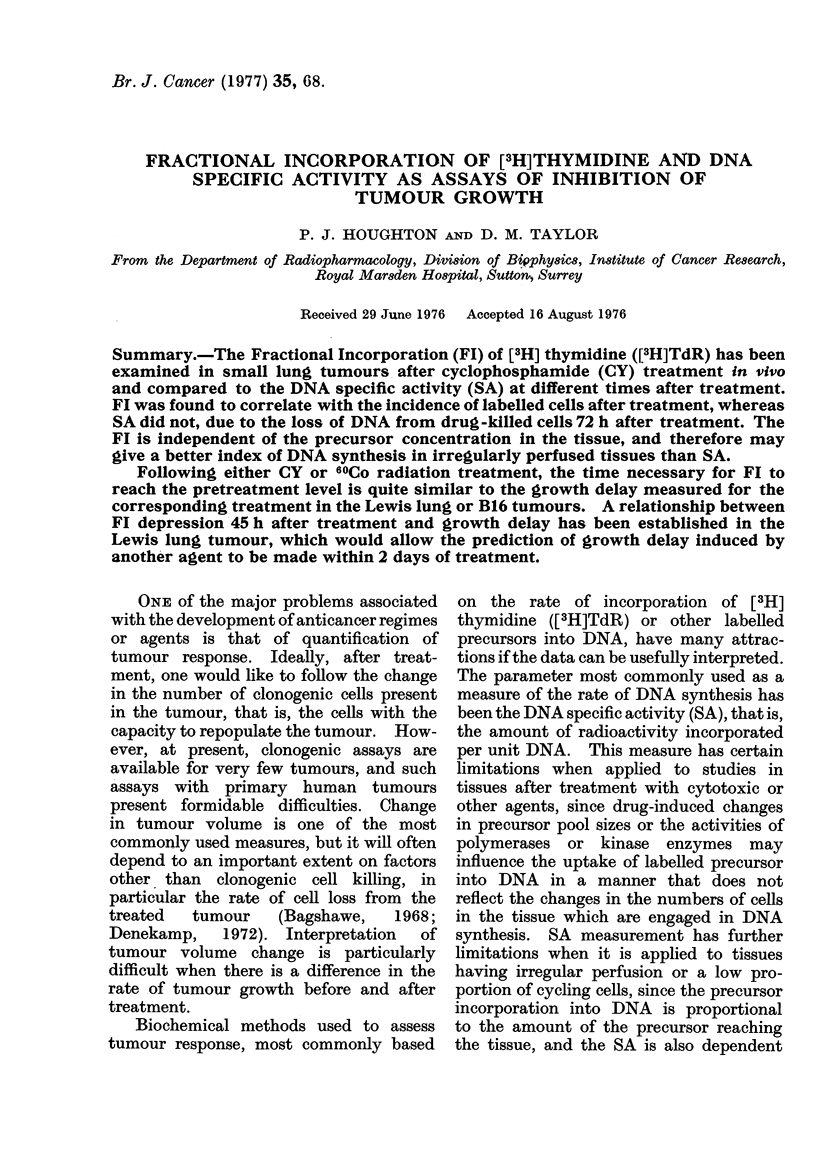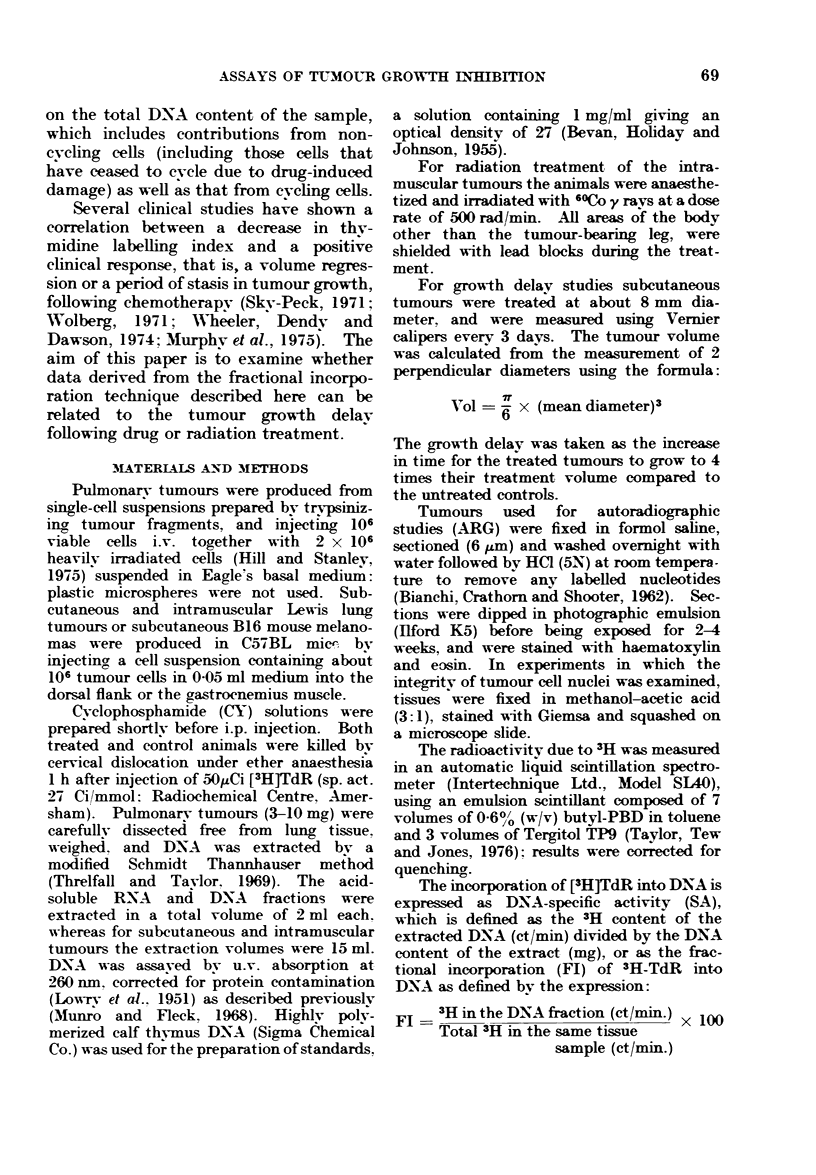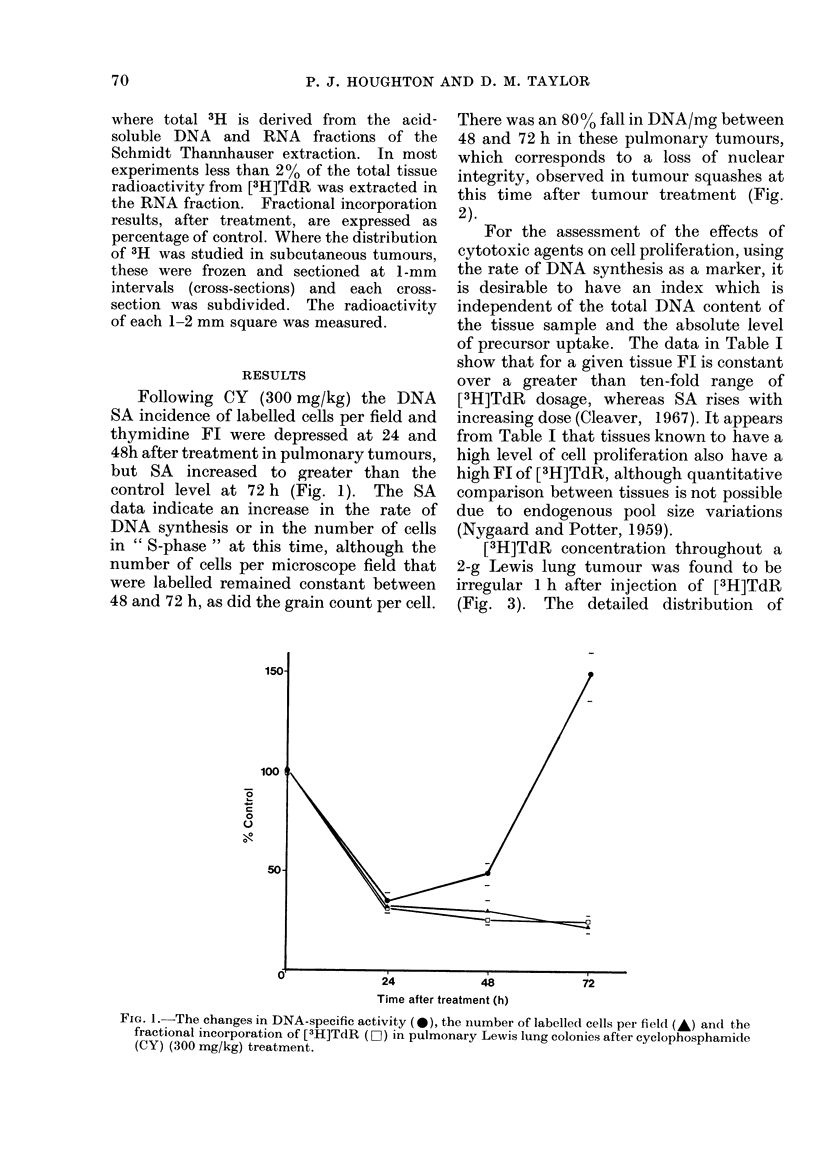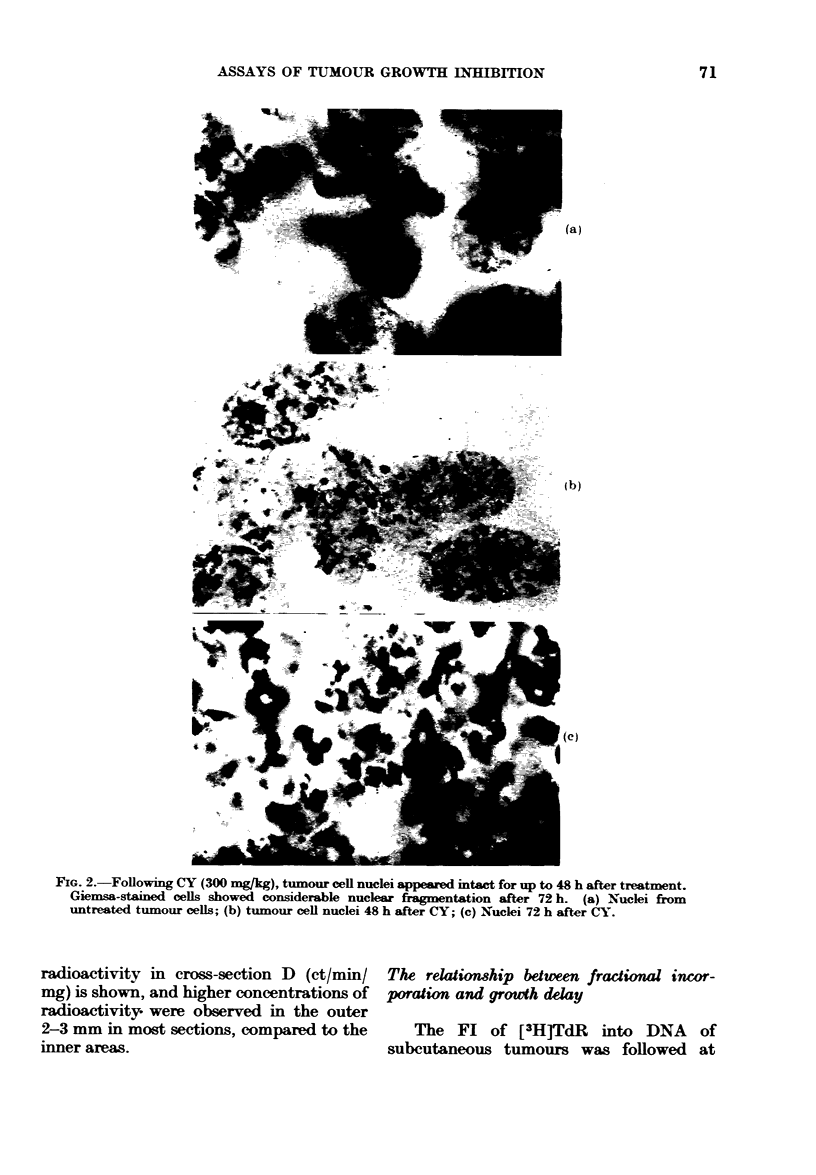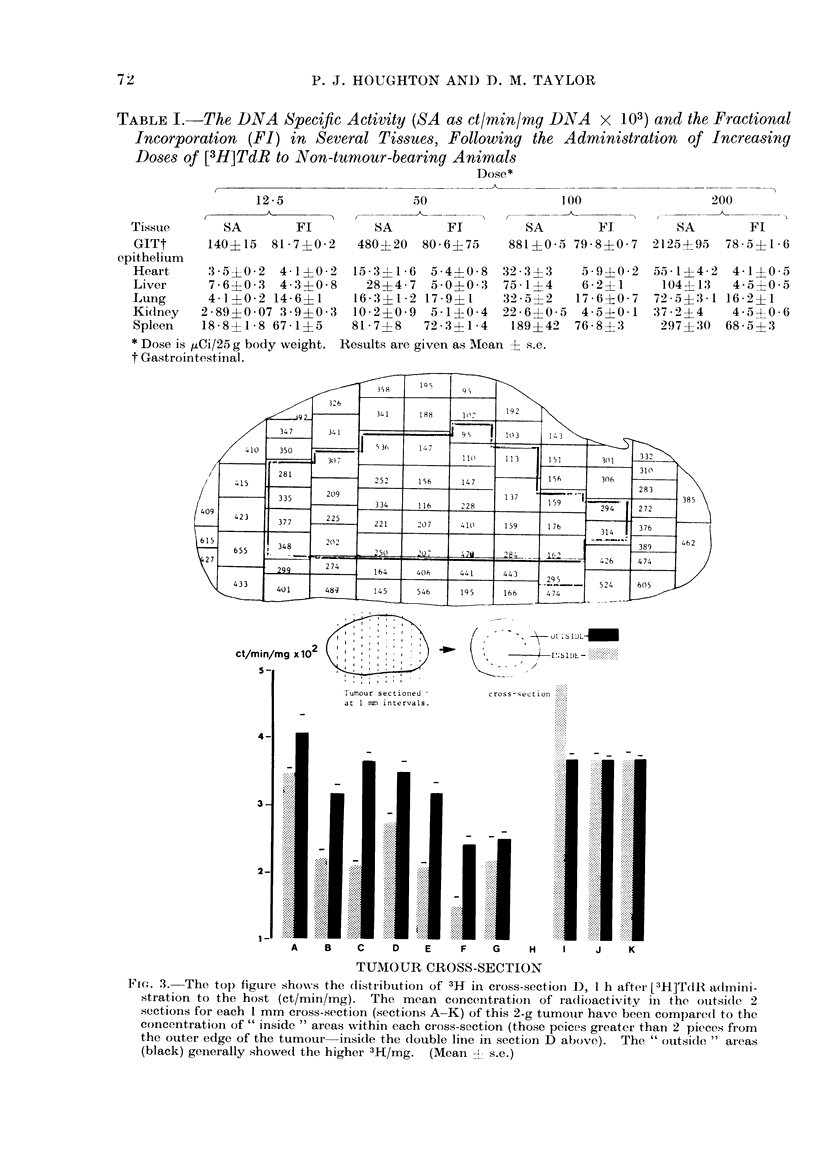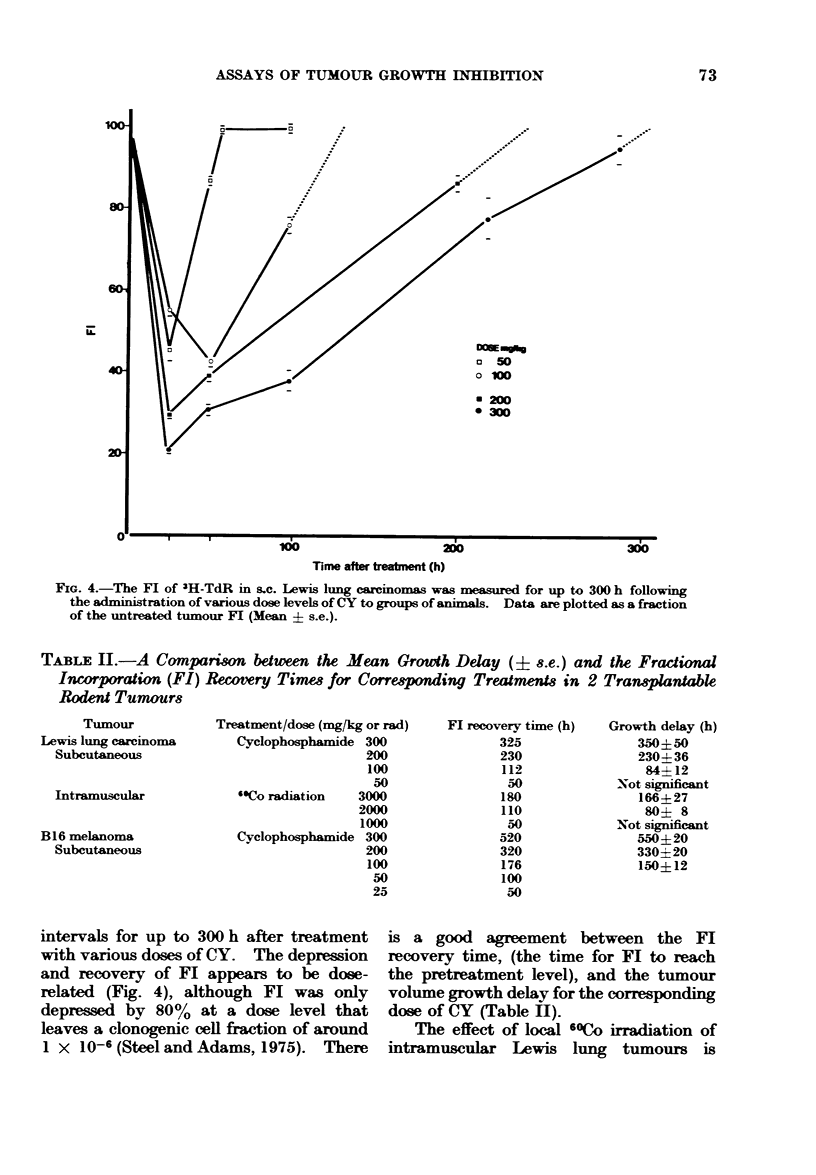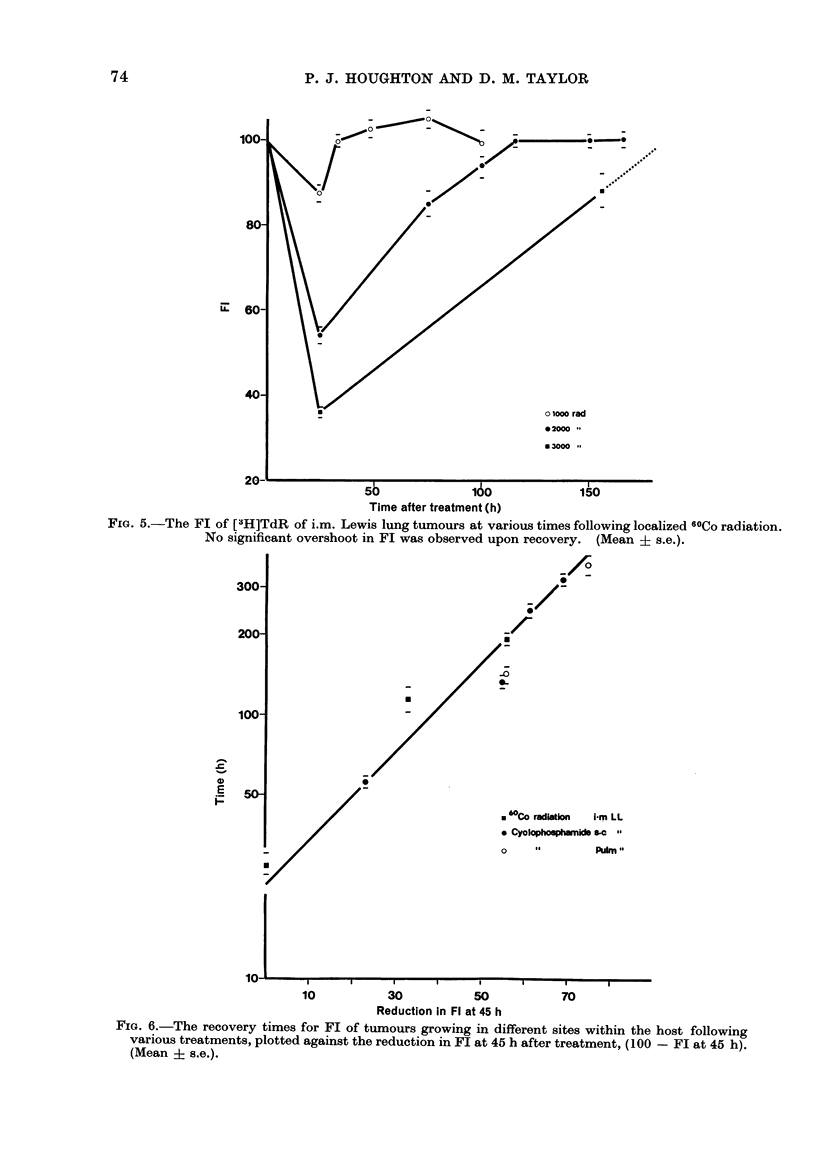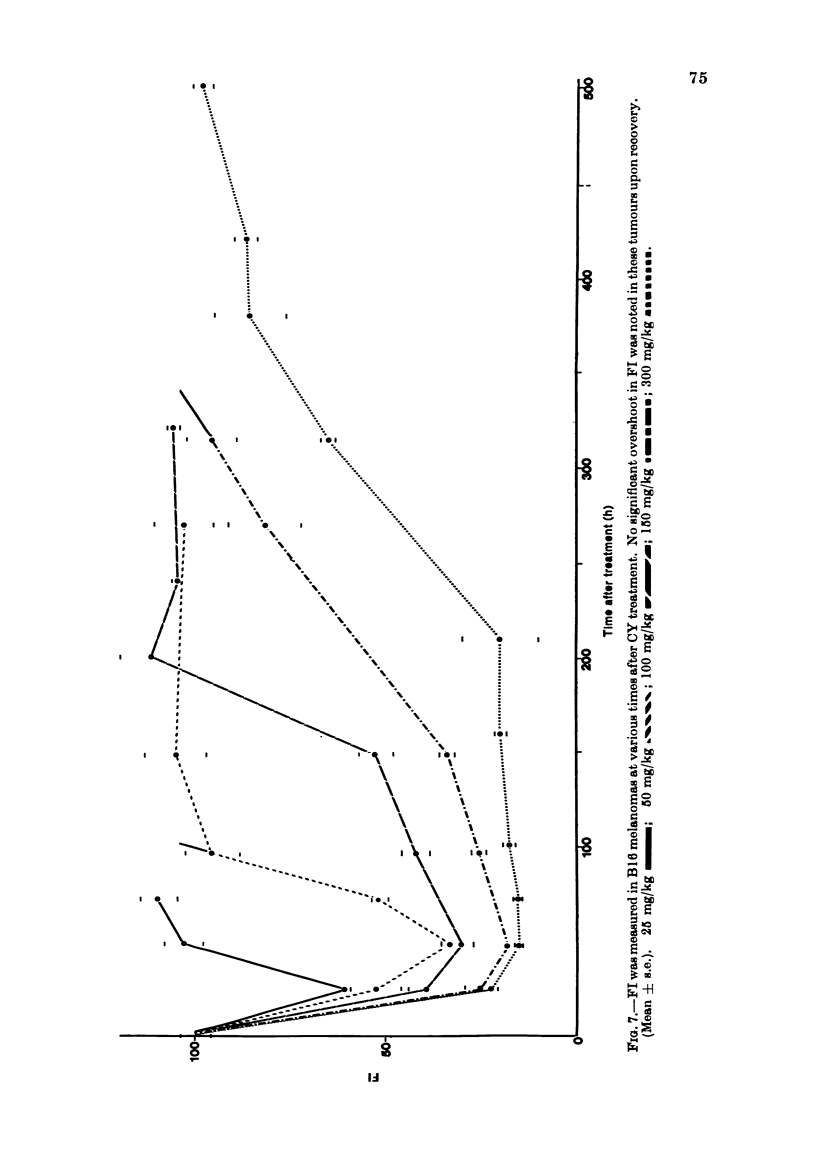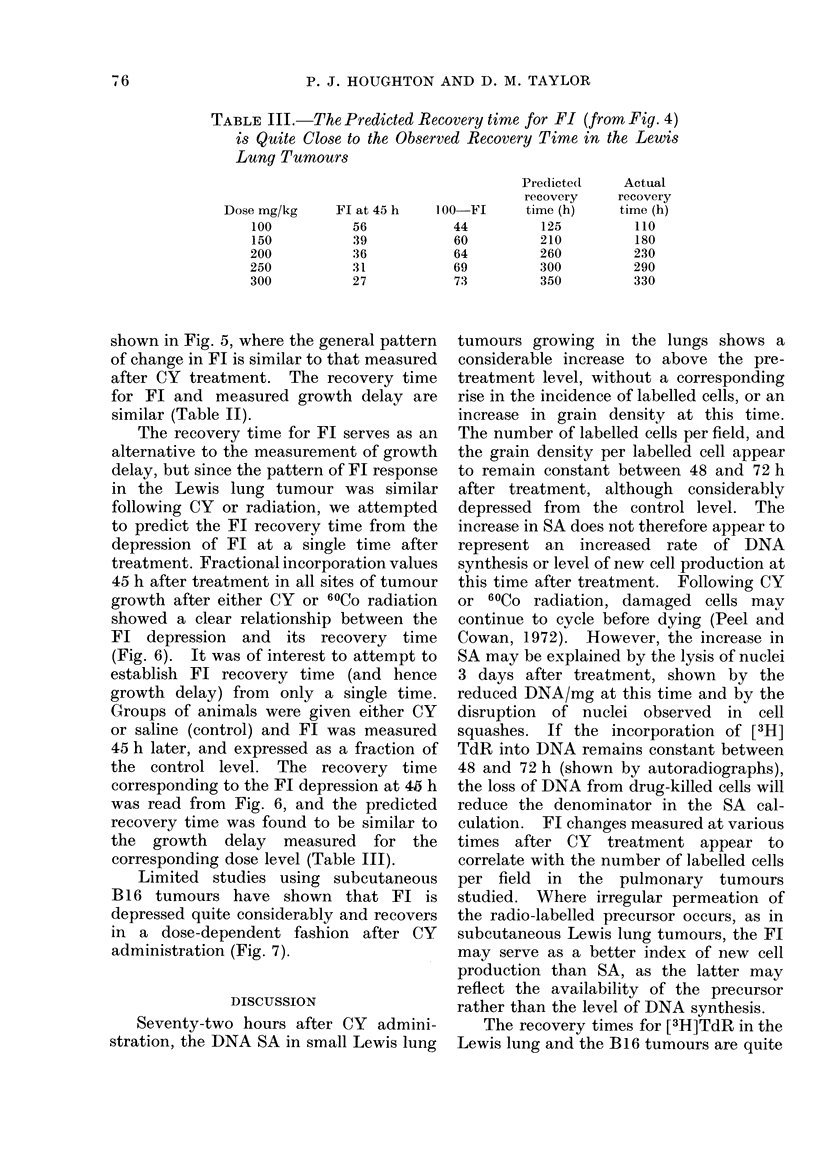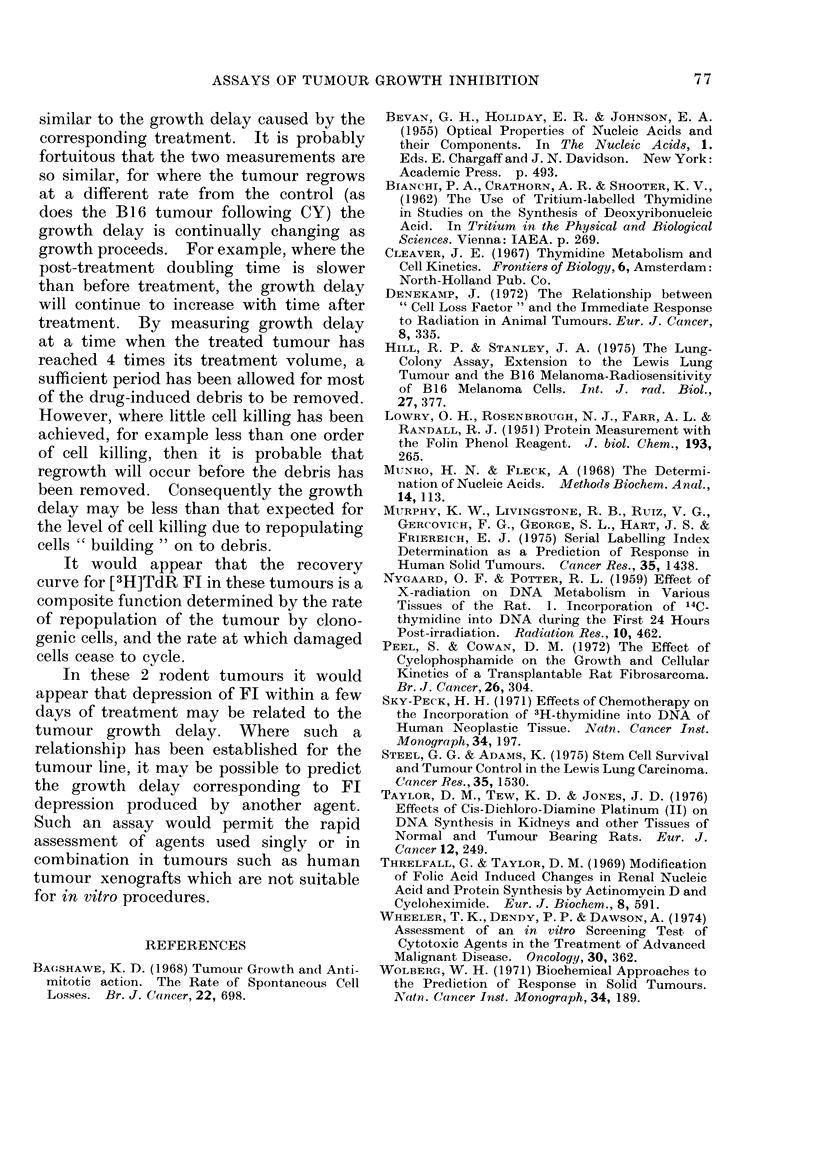# Fractional incorporation of [3H]thymidine and DNA specific activity as assays of inhibition of tumour growth.

**DOI:** 10.1038/bjc.1977.5

**Published:** 1977-01

**Authors:** P. J. Houghton, D. M. Taylor

## Abstract

**Images:**


*Br. J. Cancer* (1977) **35,** 68.

### FRACTIONAL INCORPORATION OF [$^3$H]THYMIDINE AND DNA SPECIFIC ACTIVITY AS ASSAYS OF INHIBITION OF TUMOUR GROWTH

P. J. HOUGHTON AND D. M. TAYLOR

*From the Department of Radiopharmacology, Division of Biophysics, Institute of Cancer Research, Royal Marsden Hospital, Sutton, Surrey*

Received 29 June 1976 Accepted 16 August 1976

**Summary.—The Fractional Incorporation (FI) of [$^3$H] thymidine ([$^3$H]TdR) has been examined in small lung tumours after cyclophosphamide (CY) treatment *in vivo* and compared to the DNA specific activity (SA) at different times after treatment. FI was found to correlate with the incidence of labelled cells after treatment, whereas SA did not, due to the loss of DNA from drug-killed cells 72 h after treatment. The FI is independent of the precursor concentration in the tissue, and therefore may give a better index of DNA synthesis in irregularly perfused tissues than SA.**

**Following either CY or $^{60}$Co radiation treatment, the time necessary for FI to reach the pretreatment level is quite similar to the growth delay measured for the corresponding treatment in the Lewis lung or B16 tumours. A relationship between FI depression 45 h after treatment and growth delay has been established in the Lewis lung tumour, which would allow the prediction of growth delay induced by another agent to be made within 2 days of treatment.**

One of the major problems associated with the development of anticancer regimes or agents is that of quantification of tumour response. Ideally, after treatment, one would like to follow the change in the number of clonogenic cells present in the tumour, that is, the cells with the capacity to repopulate the tumour. However, at present, clonogenic assays are available for very few tumours, and such assays with primary human tumours present formidable difficulties. Change in tumour volume is one of the most commonly used measures, but it will often depend to an important extent on factors other than clonogenic cell killing, in particular the rate of cell loss from the treated tumour (Bagshawe, 1968; Denekamp, 1972). Interpretation of tumour volume change is particularly difficult when there is a difference in the rate of tumour growth before and after treatment.

Biochemical methods used to assess tumour response, most commonly based on the rate of incorporation of [$^3$H] thymidine ([$^3$H]TdR) or other labelled precursors into DNA, have many attractions if the data can be usefully interpreted. The parameter most commonly used as a measure of the rate of DNA synthesis has been the DNA specific activity (SA), that is, the amount of radioactivity incorporated per unit DNA. This measure has certain limitations when applied to studies in tissues after treatment with cytotoxic or other agents, since drug-induced changes in precursor pool sizes or the activities of polymerases or kinase enzymes may influence the uptake of labelled precursor into DNA in a manner that does not reflect the changes in the numbers of cells in the tissue which are engaged in DNA synthesis. SA measurement has further limitations when it is applied to tissues having irregular perfusion or a low proportion of cycling cells, since the precursor incorporation into DNA is proportional to the amount of the precursor reaching the tissue, and the SA is also dependent on the total DNA content of the sample, which includes contributions from non-cycling cells (including those cells that have ceased to cycle due to drug-induced damage) as well as that from cycling cells.

Several clinical studies have shown a correlation between a decrease in thymidine labelling index and a positive clinical response, that is, a volume regression or a period of stasis in tumour growth, following chemotherapy (Sky-Peck, 1971; Wolberg, 1971; Wheeler, Dendy and Dawson, 1974; Murphy *et al.*, 1975). The aim of this paper is to examine whether data derived from the fractional incorporation technique described here can be related to the tumour growth delay following drug or radiation treatment.

### MATERIALS AND METHODS

Pulmonary tumours were produced from single-cell suspensions prepared by trypsinizing tumour fragments, and injecting $10^6$ viable cells i.v. together with $2 \times 10^6$ heavily irradiated cells (Hill and Stanley, 1975) suspended in Eagle's basal medium: plastic microspheres were not used. Subcutaneous and intramuscular Lewis lung tumours or subcutaneous B16 mouse melanomas were produced in C57BL mice by injecting a cell suspension containing about $10^6$ tumour cells in 0·05 ml medium into the dorsal flank or the gastrocnemius muscle.

Cyclophosphamide (CY) solutions were prepared shortly before i.p. injection. Both treated and control animals were killed by cervical dislocation under ether anaesthesia 1 h after injection of 50$\mu$Ci [$^3$H]TdR (sp. act. 27 Ci/mmol: Radiochemical Centre, Amersham). Pulmonary tumours (3–10 mg) were carefully dissected free from lung tissue, weighed, and DNA was extracted by a modified Schmidt Thannhauser method (Threlfall and Taylor, 1969). The acid-soluble RNA and DNA fractions were extracted in a total volume of 2 ml each, whereas for subcutaneous and intramuscular tumours the extraction volumes were 15 ml. DNA was assayed by u.v. absorption at 260 nm, corrected for protein contamination (Lowry *et al.*, 1951) as described previously (Munro and Fleck, 1968). Highly polymerized calf thymus DNA (Sigma Chemical Co.) was used for the preparation of standards, a solution containing 1 mg/ml giving an optical density of 27 (Bevan, Holiday and Johnson, 1955).

For radiation treatment of the intramuscular tumours the animals were anaesthetized and irradiated with $^{60}$Co $\gamma$ rays at a dose rate of 500 rad/min. All areas of the body other than the tumour-bearing leg, were shielded with lead blocks during the treatment.

For growth delay studies subcutaneous tumours were treated at about 8 mm diameter, and were measured using Vernier calipers every 3 days. The tumour volume was calculated from the measurement of 2 perpendicular diameters using the formula:

$$\text{Vol} = \frac{\pi}{6} \times (\text{mean diameter})^3$$

The growth delay was taken as the increase in time for the treated tumours to grow to 4 times their treatment volume compared to the untreated controls.

Tumours used for autoradiographic studies (ARG) were fixed in formol saline, sectioned (6 $\mu$m) and washed overnight with water followed by HCl (5N) at room temperature to remove any labelled nucleotides (Bianchi, Crathorn and Shooter, 1962). Sections were dipped in photographic emulsion (Ilford K5) before being exposed for 2–4 weeks, and were stained with haematoxylin and eosin. In experiments in which the integrity of tumour cell nuclei was examined, tissues were fixed in methanol–acetic acid (3:1), stained with Giemsa and squashed on a microscope slide.

The radioactivity due to $^3$H was measured in an automatic liquid scintillation spectrometer (Intertechnique Ltd., Model SL40), using an emulsion scintillant composed of 7 volumes of 0·6% (w/v) butyl-PBD in toluene and 3 volumes of Tergitol TP9 (Taylor, Tew and Jones, 1976): results were corrected for quenching.

The incorporation of [$^3$H]TdR into DNA is expressed as DNA-specific activity (SA), which is defined as the $^3$H content of the extracted DNA (ct/min) divided by the DNA content of the extract (mg), or as the fractional incorporation (FI) of $^3$H-TdR into DNA as defined by the expression:

$$\text{FI} = \frac{^3\text{H in the DNA fraction (ct/min.)}}{\text{Total }^3\text{H in the same tissue sample (ct/min.)}} \times 100$$

where total $^{3}H$ is derived from the acid-soluble DNA and RNA fractions of the Schmidt Thannhauser extraction. In most experiments less than 2% of the total tissue radioactivity from [$^{3}H$]TdR was extracted in the RNA fraction. Fractional incorporation results, after treatment, are expressed as percentage of control. Where the distribution of $^{3}H$ was studied in subcutaneous tumours, these were frozen and sectioned at 1-mm intervals (cross-sections) and each cross-section was subdivided. The radioactivity of each 1–2 mm square was measured.

### RESULTS

Following CY (300 mg/kg) the DNA SA incidence of labelled cells per field and thymidine FI were depressed at 24 and 48h after treatment in pulmonary tumours, but SA increased to greater than the control level at 72 h (Fig. 1). The SA data indicate an increase in the rate of DNA synthesis or in the number of cells in " S-phase " at this time, although the number of cells per microscope field that were labelled remained constant between 48 and 72 h, as did the grain count per cell. There was an 80% fall in DNA/mg between 48 and 72 h in these pulmonary tumours, which corresponds to a loss of nuclear integrity, observed in tumour squashes at this time after tumour treatment (Fig. 2).

For the assessment of the effects of cytotoxic agents on cell proliferation, using the rate of DNA synthesis as a marker, it is desirable to have an index which is independent of the total DNA content of the tissue sample and the absolute level of precursor uptake. The data in Table I show that for a given tissue FI is constant over a greater than ten-fold range of [$^{3}H$]TdR dosage, whereas SA rises with increasing dose (Cleaver, 1967). It appears from Table I that tissues known to have a high level of cell proliferation also have a high FI of [$^{3}H$]TdR, although quantitative comparison between tissues is not possible due to endogenous pool size variations (Nygaard and Potter, 1959).

[$^{3}H$]TdR concentration throughout a 2-g Lewis lung tumour was found to be irregular 1 h after injection of [$^{3}H$]TdR (Fig. 3). The detailed distribution of

FIG. 1.—The changes in DNA-specific activity (●), the number of labelled cells per field (▲) and the fractional incorporation of [$^{3}H$]TdR (□) in pulmonary Lewis lung colonies after cyclophosphamide (CY) (300 mg/kg) treatment.

FIG. 2.—Following CY (300 mg/kg), tumour cell nuclei appeared intact for up to 48 h after treatment. Giemsa-stained cells showed considerable nuclear fragmentation after 72 h. (a) Nuclei from untreated tumour cells; (b) tumour cell nuclei 48 h after CY; (c) Nuclei 72 h after CY.

radioactivity in cross-section D (ct/min/mg) is shown, and higher concentrations of radioactivity were observed in the outer 2–3 mm in most sections, compared to the inner areas.

*The relationship between fractional incorporation and growth delay*

The FI of [$^3$H]TdR into DNA of subcutaneous tumours was followed at

TABLE I.—*The DNA Specific Activity (SA as ct/min/mg DNA* × 10³) *and the Fractional Incorporation (FI) in Several Tissues, Following the Administration of Increasing Doses of [³H]TdR to Non-tumour-bearing Animals*

| | Dose* | | | | | | | |
|---|---|---|---|---|---|---|---|---|
| | 12·5 | | 50 | | 100 | | 200 | |
| Tissue | SA | FI | SA | FI | SA | FI | SA | FI |
| GIT† epithelium | 140±15 | 81·7±0·2 | 480±20 | 80·6±75 | 881±0·5 | 79·8±0·7 | 2125±95 | 78·5±1·6 |
| Heart | 3·5±0·2 | 4·1±0·2 | 15·3±1·6 | 5·4±0·8 | 32·3±3 | 5·9±0·2 | 55·1±4·2 | 4·1±0·5 |
| Liver | 7·6±0·3 | 4·3±0·8 | 28±4·7 | 5·0±0·3 | 75·1±4 | 6·2±1 | 104±13 | 4·5±0·5 |
| Lung | 4·1±0·2 | 14·6±1 | 16·3±1·2 | 17·9±1 | 32·5±2 | 17·6±0·7 | 72·5±3·1 | 16·2±1 |
| Kidney | 2·89±0·07 | 3·9±0·3 | 10·2±0·9 | 5·1±0·4 | 22·6±0·5 | 4·5±0·1 | 37·2±4 | 4·5±0·6 |
| Spleen | 18·8±1·8 | 67·1±5 | 81·7±8 | 72·3±1·4 | 189±42 | 76·8±3 | 297±30 | 68·5±3 |

* Dose is μCi/25 g body weight. Results are given as Mean ± s.e.

† Gastrointestinal.

FIG. 3.—The top figure shows the distribution of ³H in cross-section D, 1 h after [³H]TdR administration to the host (ct/min/mg). The mean concentration of radioactivity in the outside 2 sections for each 1 mm cross-section (sections A–K) of this 2-g tumour have been compared to the concentration of " inside " areas within each cross-section (those peices greater than 2 pieces from the outer edge of the tumour—inside the double line in section D above). The " outside " areas (black) generally showed the higher ³H/mg. (Mean ± s.e.)

FIG. 4.—The FI of $^3$H-TdR in s.c. Lewis lung carcinomas was measured for up to 300 h following the administration of various dose levels of CY to groups of animals. Data are plotted as a fraction of the untreated tumour FI (Mean ± s.e.).

TABLE II.—*A Comparison between the Mean Growth Delay (± s.e.) and the Fractional Incorporation (FI) Recovery Times for Corresponding Treatments in 2 Transplantable Rodent Tumours*

| Tumour | Treatment/dose (mg/kg or rad) | | FI recovery time (h) | Growth delay (h) |
|---|---|---|---|---|
| Lewis lung carcinoma | Cyclophosphamide | 300 | 325 | 350±50 |
| Subcutaneous | | 200 | 230 | 230±36 |
| | | 100 | 112 | 84±12 |
| | | 50 | 50 | Not significant |
| Intramuscular | $^{60}$Co radiation | 3000 | 180 | 166±27 |
| | | 2000 | 110 | 80± 8 |
| | | 1000 | 50 | Not significant |
| B16 melanoma | Cyclophosphamide | 300 | 520 | 550±20 |
| Subcutaneous | | 200 | 320 | 330±20 |
| | | 100 | 176 | 150±12 |
| | | 50 | 100 | |
| | | 25 | 50 | |

intervals for up to 300 h after treatment with various doses of CY. The depression and recovery of FI appears to be dose-related (Fig. 4), although FI was only depressed by 80% at a dose level that leaves a clonogenic cell fraction of around $1 \times 10^{-6}$ (Steel and Adams, 1975). There is a good agreement between the FI recovery time, (the time for FI to reach the pretreatment level), and the tumour volume growth delay for the corresponding dose of CY (Table II).

The effect of local $^{60}$Co irradiation of intramuscular Lewis lung tumours is

FIG. 5.—The FI of [³H]TdR of i.m. Lewis lung tumours at various times following localized $^{60}Co$ radiation. No significant overshoot in FI was observed upon recovery. (Mean ± s.e.).

FIG. 6.—The recovery times for FI of tumours growing in different sites within the host following various treatments, plotted against the reduction in FI at 45 h after treatment, (100 − FI at 45 h). (Mean ± s.e.).

FIG. 7.—FI was measured in B16 melanomas at various times after CY treatment. No significant overshoot in FI was noted in these tumours upon recovery. (Mean ± s.e.). 25 mg/kg ▬▬▬; 50 mg/kg ╲╲╲╲; 100 mg/kg ▬▬▬; 150 mg/kg ▪▬▪▬▪; 300 mg/kg ▪▪▪▪▪▪▪.

TABLE III.—*The Predicted Recovery time for FI (from Fig. 4) is Quite Close to the Observed Recovery Time in the Lewis Lung Tumours*

| Dose mg/kg | FI at 45 h | 100—FI | Predicted recovery time (h) | Actual recovery time (h) |
|---|---|---|---|---|
| 100 | 56 | 44 | 125 | 110 |
| 150 | 39 | 60 | 210 | 180 |
| 200 | 36 | 64 | 260 | 230 |
| 250 | 31 | 69 | 300 | 290 |
| 300 | 27 | 73 | 350 | 330 |

shown in Fig. 5, where the general pattern of change in FI is similar to that measured after CY treatment. The recovery time for FI and measured growth delay are similar (Table II).

The recovery time for FI serves as an alternative to the measurement of growth delay, but since the pattern of FI response in the Lewis lung tumour was similar following CY or radiation, we attempted to predict the FI recovery time from the depression of FI at a single time after treatment. Fractional incorporation values 45 h after treatment in all sites of tumour growth after either CY or $^{60}$Co radiation showed a clear relationship between the FI depression and its recovery time (Fig. 6). It was of interest to attempt to establish FI recovery time (and hence growth delay) from only a single time. Groups of animals were given either CY or saline (control) and FI was measured 45 h later, and expressed as a fraction of the control level. The recovery time corresponding to the FI depression at 45 h was read from Fig. 6, and the predicted recovery time was found to be similar to the growth delay measured for the corresponding dose level (Table III).

Limited studies using subcutaneous B16 tumours have shown that FI is depressed quite considerably and recovers in a dose-dependent fashion after CY administration (Fig. 7).

### DISCUSSION

Seventy-two hours after CY administration, the DNA SA in small Lewis lung tumours growing in the lungs shows a considerable increase to above the pre-treatment level, without a corresponding rise in the incidence of labelled cells, or an increase in grain density at this time. The number of labelled cells per field, and the grain density per labelled cell appear to remain constant between 48 and 72 h after treatment, although considerably depressed from the control level. The increase in SA does not therefore appear to represent an increased rate of DNA synthesis or level of new cell production at this time after treatment. Following CY or $^{60}$Co radiation, damaged cells may continue to cycle before dying (Peel and Cowan, 1972). However, the increase in SA may be explained by the lysis of nuclei 3 days after treatment, shown by the reduced DNA/mg at this time and by the disruption of nuclei observed in cell squashes. If the incorporation of [$^3$H] TdR into DNA remains constant between 48 and 72 h (shown by autoradiographs), the loss of DNA from drug-killed cells will reduce the denominator in the SA calculation. FI changes measured at various times after CY treatment appear to correlate with the number of labelled cells per field in the pulmonary tumours studied. Where irregular permeation of the radio-labelled precursor occurs, as in subcutaneous Lewis lung tumours, the FI may serve as a better index of new cell production than SA, as the latter may reflect the availability of the precursor rather than the level of DNA synthesis.

The recovery times for [$^3$H]TdR in the Lewis lung and the B16 tumours are quite similar to the growth delay caused by the corresponding treatment. It is probably fortuitous that the two measurements are so similar, for where the tumour regrows at a different rate from the control (as does the B16 tumour following CY) the growth delay is continually changing as growth proceeds. For example, where the post-treatment doubling time is slower than before treatment, the growth delay will continue to increase with time after treatment. By measuring growth delay at a time when the treated tumour has reached 4 times its treatment volume, a sufficient period has been allowed for most of the drug-induced debris to be removed. However, where little cell killing has been achieved, for example less than one order of cell killing, then it is probable that regrowth will occur before the debris has been removed. Consequently the growth delay may be less than that expected for the level of cell killing due to repopulating cells " building " on to debris.

It would appear that the recovery curve for [$^3$H]TdR FI in these tumours is a composite function determined by the rate of repopulation of the tumour by clonogenic cells, and the rate at which damaged cells cease to cycle.

In these 2 rodent tumours it would appear that depression of FI within a few days of treatment may be related to the tumour growth delay. Where such a relationship has been established for the tumour line, it may be possible to predict the growth delay corresponding to FI depression produced by another agent. Such an assay would permit the rapid assessment of agents used singly or in combination in tumours such as human tumour xenografts which are not suitable for *in vitro* procedures.

### REFERENCES

BAGSHAWE, K. D. (1968) Tumour Growth and Antimitotic action. The Rate of Spontaneous Cell Losses. *Br. J. Cancer*, **22**, 698.

BEVAN, G. H., HOLIDAY, E. R. & JOHNSON, E. A. (1955) Optical Properties of Nucleic Acids and their Components. In *The Nucleic Acids*, **1.** Eds. E. Chargaff and J. N. Davidson. New York: Academic Press. p. 493.

BIANCHI, P. A., CRATHORN, A. R. & SHOOTER, K. V., (1962) The Use of Tritium-labelled Thymidine in Studies on the Synthesis of Deoxyribonucleic Acid. In *Tritium in the Physical and Biological Sciences.* Vienna: IAEA. p. 269.

CLEAVER, J. E. (1967) Thymidine Metabolism and Cell Kinetics. *Frontiers of Biology*, **6**, Amsterdam: North-Holland Pub. Co.

DENEKAMP, J. (1972) The Relationship between " Cell Loss Factor " and the Immediate Response to Radiation in Animal Tumours. *Eur. J. Cancer*, **8**, 335.

HILL, R. P. & STANLEY, J. A. (1975) The Lung-Colony Assay, Extension to the Lewis Lung Tumour and the B16 Melanoma-Radiosensitivity of B16 Melanoma Cells. *Int. J. rad. Biol.*, **27**, 377.

LOWRY, O. H., ROSENBROUGH, N. J., FARR, A. L. & RANDALL, R. J. (1951) Protein Measurement with the Folin Phenol Reagent. *J. biol. Chem.*, **193**, 265.

MUNRO, H. N. & FLECK, A (1968) The Determination of Nucleic Acids. *Methods Biochem. Anal.*, **14**, 113.

MURPHY, K. W., LIVINGSTONE, R. B., RUIZ, V. G., GERCOVICH, F. G., GEORGE, S. L., HART, J. S. & FRIEREICH, E. J. (1975) Serial Labelling Index Determination as a Prediction of Response in Human Solid Tumours. *Cancer Res.*, **35**, 1438.

NYGAARD, O. F. & POTTER, R. L. (1959) Effect of X-radiation on DNA Metabolism in Various Tissues of the Rat. 1. Incorporation of $^{14}$C-thymidine into DNA during the First 24 Hours Post-irradiation. *Radiation Res.*, **10**, 462.

PEEL, S. & COWAN, D. M. (1972) The Effect of Cyclophosphamide on the Growth and Cellular Kinetics of a Transplantable Rat Fibrosarcoma. *Br. J. Cancer*, **26**, 304.

SKY-PECK, H. H. (1971) Effects of Chemotherapy on the Incorporation of $^3$H-thymidine into DNA of Human Neoplastic Tissue. *Natn. Cancer Inst. Monograph*, **34**, 197.

STEEL, G. G. & ADAMS, K. (1975) Stem Cell Survival and Tumour Control in the Lewis Lung Carcinoma. *Cancer Res.*, **35**, 1530.

TAYLOR, D. M., TEW, K. D. & JONES, J. D. (1976) Effects of Cis-Dichloro-Diamine Platinum (II) on DNA Synthesis in Kidneys and other Tissues of Normal and Tumour Bearing Rats. *Eur. J. Cancer* **12**, 249.

THRELFALL, G. & TAYLOR, D. M. (1969) Modification of Folic Acid Induced Changes in Renal Nucleic Acid and Protein Synthesis by Actinomycin D and Cycloheximide. *Eur. J. Biochem.*, **8**, 591.

WHEELER, T. K., DENDY, P. P. & DAWSON, A. (1974) Assessment of an *in vitro* Screening Test of Cytotoxic Agents in the Treatment of Advanced Malignant Disease. *Oncology*, **30**, 362.

WOLBERG, W. H. (1971) Biochemical Approaches to the Prediction of Response in Solid Tumours. *Natn. Cancer Inst. Monograph*, **34**, 189.